# CATCH-ON Age-Friendly Health System Implementation Across Varied Geographic and System Settings

**DOI:** 10.1093/geroni/igaa057.2590

**Published:** 2020-12-16

**Authors:** Erin Emery-Tiburcio, Robyn Golden, Michelle Newman

**Affiliations:** 1 Rush University Medical Center, Chicago, Illinois, United States; 2 Rush University Medical Center, CHICAGO, Illinois, United States

## Abstract

GWEP program goals include transformation of clinical training environments into integrated geriatrics and primary care systems to become Age-Friendly Health Systems. CATCH-ON, the collaborative GWEP led by Rush University Medical Center, is working with primary care health systems in four highly varied geographic and system settings to achieve this goal. Each of these clinic systems has unique successes and challenges in developing and implementing workflow protocols; engaging providers, patients, families, and community-based organizations in the development of Age-Friendly Health Communities; and modifying the electronic health record to document assessing and acting on 4Ms. Clinic team members participate in monthly Learning Community sessions to learn about and reinforce the 4Ms, along with practical recommendations for implementing with patients. This session will focus on CATCH-ON’s process for implementing the 4Ms in various practice settings, lessons learned in implementation across large and small health systems, and opportunities to bridge AFHS with the community.

